# Comparison of Mechanical Characteristics of Three Different Types of Wires for Bonded Lingual Retainer Fabrication

**DOI:** 10.30476/dentjods.2025.106705.2684

**Published:** 2026-09-01

**Authors:** Fatemeh Keikhaee, Neda Shahraki, Hedie Mohsenzade

**Affiliations:** 1 Speciality in Orthodontics, Dept. of Orthodontics, Faculty of Dentistry, Zahedan University of Medical Sciences, Zahedan, Iran.

**Keywords:** Orthodontic Retainers, Orthodontic, Bone Wires, Orthodontic Wires, Orthodontic Appliances

## Abstract

**Background:**

Bonded lingual retainers (BLRs) are essential in orthodontics to maintain post-treatment alignment, particularly for mandibular and maxillary anterior teeth. The mechanical properties of the wires used in BLRs significantly influence their performance and longevity.

**Purpose:**

This study evaluates the mechanical characteristics of three wire types commonly used in BLRs including Coaxial, Dead Soft, and Multi-Strand Retainer wires.

**Materials and Method:**

This *in vitro* study was description-analytical. A total of 120 extracted human premolar teeth (60 dental double blocks and 60 acrylic blocks) were divided into three groups based on wire type. Teeth were prepared using standard etching and bonding procedures, followed by wire placement and curing. Mechanical tests, including detachment force evaluation, fracture mode analysis, deformation measurement, and pull-out testing were conducted using an Instron Testing Machine. Statistical analysis was performed using SPSS software (version 23.0), employing ANOVA and non-parametric tests where appropriate.

**Results:**

The Dead Soft wire exhibited the highest pull-out force (92.92 N) and detachment force (51.68 N), while the Multi-Strand Retainer wire showed the lowest pull-out force (58.38 N). The Coaxial wire demonstrated the highest deformation (1.34mm), whereas the Dead Soft wire had the least deformation (0.91mm). Fracture mode analysis revealed significant differences among groups; Coaxial wires predominantly exhibited type 3 fractures (90%), while Dead Soft wires had no type 3 fractures.

**Conclusion:**

The Dead Soft wire outperformed other wire types in terms of pull-out and detachment forces, making it a robust choice for BLRs. However, its lower deformation may reduce flexibility under stress. These findings provide valuable insights for orthodontists in selecting optimal wires for BLR fabrication to enhance clinical outcomes and device longevity.

## Introduction

Numerous orthodontists contend that as the length of the dental arch diminishes and the crowding of the lower anterior teeth escalates over time, the sole method to preserve optimal alignment following orthodontic treatment is through a type of permanent retention [ 1 - 2 ]. Permanent or long-term fixed retention is a crucial aspect of orthodontic treatment, as it has been shown to preserve the stability of the achieved results [ 3 ]. In orthodontic practice, bonded retainers are commonly placed on the lingual surfaces of the mandibular and frequently the maxillary anterior teeth, serving as a fundamental element of treatment protocols [ 4 ].

The choice of wires for bonded lingual retainers (BLRs) is essential for achieving optimal functionality and aesthetic quality [ 5 ]. A range of wire types has been developed, each possessing unique mechanical characteristics and specific uses within orthodontic practice [ 6 - 7 ]. There are two primary types of mandibular fixed wire retainers: (1) round, rigid stainless steel wires which are bonded solely to the canines and are known as canine-and-canine retainers, and (2) canine-to-canine retainers, which are made from smaller cross-section multistranded round wires that are bonded to all anterior teeth. Alongside the conventional wire retainers, alternatives such as fiber-reinforced materials and alumina ceramic retainers have also been introduced [ 8 - 9 ].

A variety of wires is available for BLRs, each exhibiting unique mechanical properties that can influence their performance [ 10 ]. Notably, three wire types are particularly prominent including (1) PentaOne, which is a five-stranded coaxial wire, and (2) Bond-A-Braid, an eight-braided dead-soft wire, and (3) Respond, a dead-soft coaxial wire. Each of these wire types offers specific benefits and drawbacks concerning strength, flexibility, and deformation when subjected to stress [ 11 - 12 ].

It is crucial for orthodontists to comprehend the mechanical attributes of these wires to make well-informed choices regarding their application in clinical settings [ 13 ]. Given that BLRs are designed for prolonged use within the oral cavity, it is essential to enhance the success rate of these devices [ 14 ]. Consequently, the selection of wire may play a significant role in optimizing the effectiveness of lingual retainers. Accordingly, the aim of this study was to comparison of mechanical characteristics of three different types of wires, focusing on detachment force, deformation, fracture mode, and pull-out force for BLR fabrication.

## Materials and Method

This *in vitro* study was conducted in accordance with ethical principles and guidelines for research involving human subjects. All procedures were approved by the Ethics Committee of Zahedan University of Medical Sciences. Informed consent was obtained from all patients prior to the collection of dental specimens used in this research. Confidentiality of patient information was strictly maintained throughout the study. Furthermore, all efforts were made to minimize discomfort or risk to participants, and all dental procedures were performed as part of routine clinical care.

The sample size was determined based on a priori power analysis conducted using G*Power software (version 3.1, Universitat Düsseldorf, Germany). An effect size (f) of 0.4 was selected based on previously reported differences in mechanical properties among various types of orthodontic wires. The significance level (α) was set at 0.05 to maintain a standard threshold for Type I error probability, ensuring that any observed differences were statistically valid. A desired statistical power (1−β) of 80% was chosen, meaning there was an 80% chance of detecting a true effect if one existed, thereby minimizing the risk of Type II error. Based on these parameters, the power analysis indicated that a minimum of 16 specimens per group would be necessary to achieve sufficient statistical power. Totally, in this study, 120 human first and second premolar teeth were collected from patients receiving dental orthodontics. Teeth with caries, cracks, or other abnormalities were excluded from the study. Residual soft tissue was removed using a scaler, and the teeth were preserved in a thymol solution so we had 60 dental double blocks, including 20 dental double blocks with teeth per group (total of 60 dental double blocks with teeth) and 20 blocks without teeth per group (total of 60 blocks without teeth). 

Acrylic resin was placed in plastic molds and the roots of the teeth were embedded in acrylic. The roots were positioned such that the long-axes of the teeth were oriented at a right angle to the base of the moA total of 60 dental double blocks were created and each of these blocks was divided into three groups containing 20 dental blocks for further procedure. The lingual surface of the teeth was polished with pumice paste without fluoride. In the next step, it was etched with 37% orthophosphoric acid and washed for 30 seconds and then dried. Then bonding (Amber FGM, Joinville, Brazil) was applied according to the manufacturer's instructions and cured with the LED device (Kerr Corporation, Orange, CA, USA).

To ensure optimal adaptation of the wire to the tooth surface, a gentle curve was applied. The first 60 dental double blocks were categorized into three distinct groups, with each group receiving a different type of wire. The specific wires utilized for each group were as Group AI: 0.175-inch Coaxial wire (American Orthodontics, USA), Group AII: 0.175-inch three-stranded wire (Multi Strand; American Orthodontics, USA), and Group AIII: 0.010 * 0.32-inch dead-soft coaxial wire (American Orthodontics, USA).

**Figure 1:**
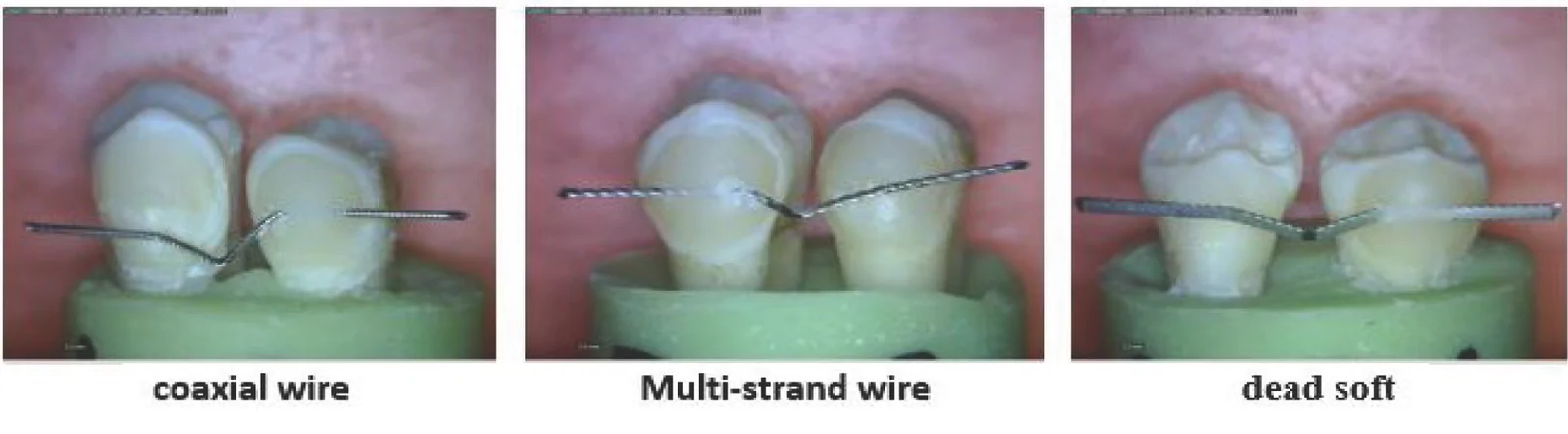
The 60 dental blocks were categorized into three distinct groups mentioned in the image

The second 60 dental double blocks were categorized into three distinct groups. In these groups, different composite wires (similar to the grouping of the previous section) were placed inside the acrylic block and subjected to the tensile test only. These groups included Group BI: 0.175-inch Coaxial wire (American Orthodontics, USA), Group BII: 0.175-inch three-stranded wire (Multi Strand; American Orthodontics, USA), and Group BIII: 0.010 * 0.32-inch dead-soft coaxial wire (American Orthodontics, USA) (Figure 1) (Supplementary Material 1). 

**Supplementary 1:** Summary of Experimental Groups and Wire Types

Group designation	Wire type	Wire dimensions	Purpose
Group A I	Coaxial	0.175-inch	Detachment Force, Deformation, Fracture Mode
Group A II	Multi-Strand Retainer	0.175-inch	Detachment Force, Deformation, Fracture Mode
Group A III	Dead Soft	0.010 * 0.32-inch	Detachment Force, Deformation, Fracture Mode
Group B I	Coaxial	0.175-inch	Pull-Out Force
Group B II	Multi-Strand Retainer	0.175-inch	Pull-Out Force
Group B III	Dead Soft	0.010 * 0.32-inch	Pull-Out Force

A segment of test wire measuring 10mm in length was cut, and its midpoint was indicated using a pencil. This test wire was subsequently positioned on the primed surface of the tooth. Great care was taken to ensure that the wire was aligned parallel to the base of the mold and situated beneath the contact point between the teeth within the mold. The composite material was then applied using a flowable composite syringe Denfil flowable composite (Vericom Co., Ltd., Gangwondo, South Korea) and cured for 10 seconds with an LED curing device. The tip of the light curing unit was positioned as closely as possible to the tooth surface. Following the curing process, the teeth were immersed in distilled water at 24°C for a period of 24 hours prior to testing .Then detachment force, fracture mode, and wire deformation tests were performed on these blocks containing teeth.

Embedded specimens were secured in a custom jig mounted to the base plate of an Instron testing machine (Instron Corp., Norwood, MA, USA).The crosshead speed was calibrated to 1mm per minute, and the maximum load required to detach the wire was documented. Following the occurrence of failure, the composite material surrounding the wire was carefully removed using a tungsten carbide bur. 

We conducted an evaluation of the fracture mode on the side where the initial bond failure was observed, utilizing an optical stereomicroscope ((SZ 40; Olympus, Tokyo, Japan)) at a magnification of 20×. The residual adhesive present on the enamel surface was assessed by a single investigator, who was blind to the treatment group assignments. The fracture sites were classified according to the adhesive remnant index (ARI). This classification system assigns scores ranging from 0 to 3, reflecting the quantity of adhesive remaining after the removal of the bracket as (0) indicates no adhesive remaining on the enamel surface, (1) signifies less than 50% adhesive remaining on the tooth, (2) denotes more than 50% adhesive remaining on the tooth, and (3) represents all adhesive still present on the tooth surface.

**Figure 2:**
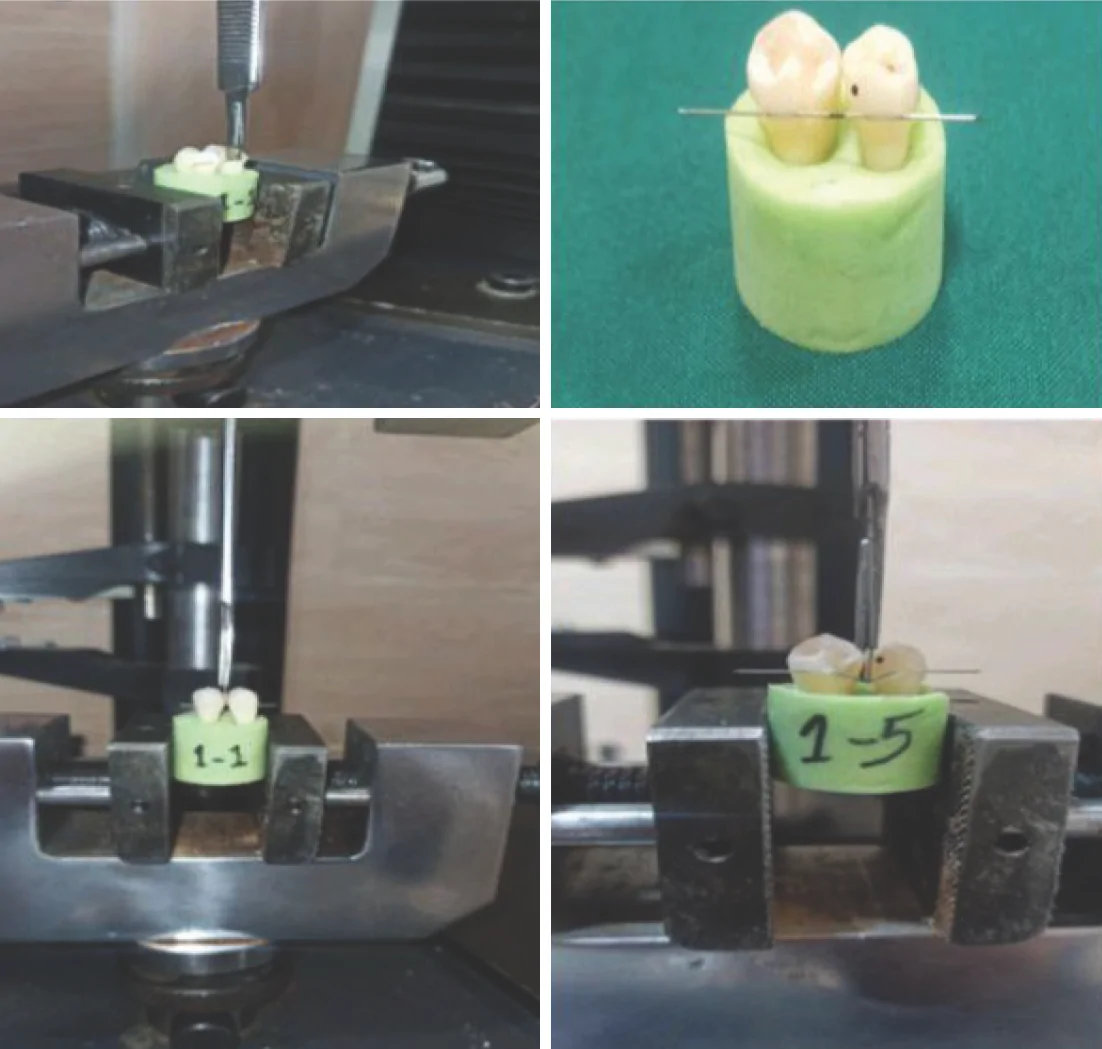
Analysis of detachment force, fracture mode, and deformation force

After fracture, the composite on the wire was gently removed with a tungsten carbide bur. Subsequently, the wire was placed on graph paper, and its deflection was evaluated under an optical stereomicroscope (SZ 40; Olympus, Tokyo, Japan) at a magnification of ×20. The deflection measurement was recorded in millimeters (Figure 2). 

**Figure 3:**
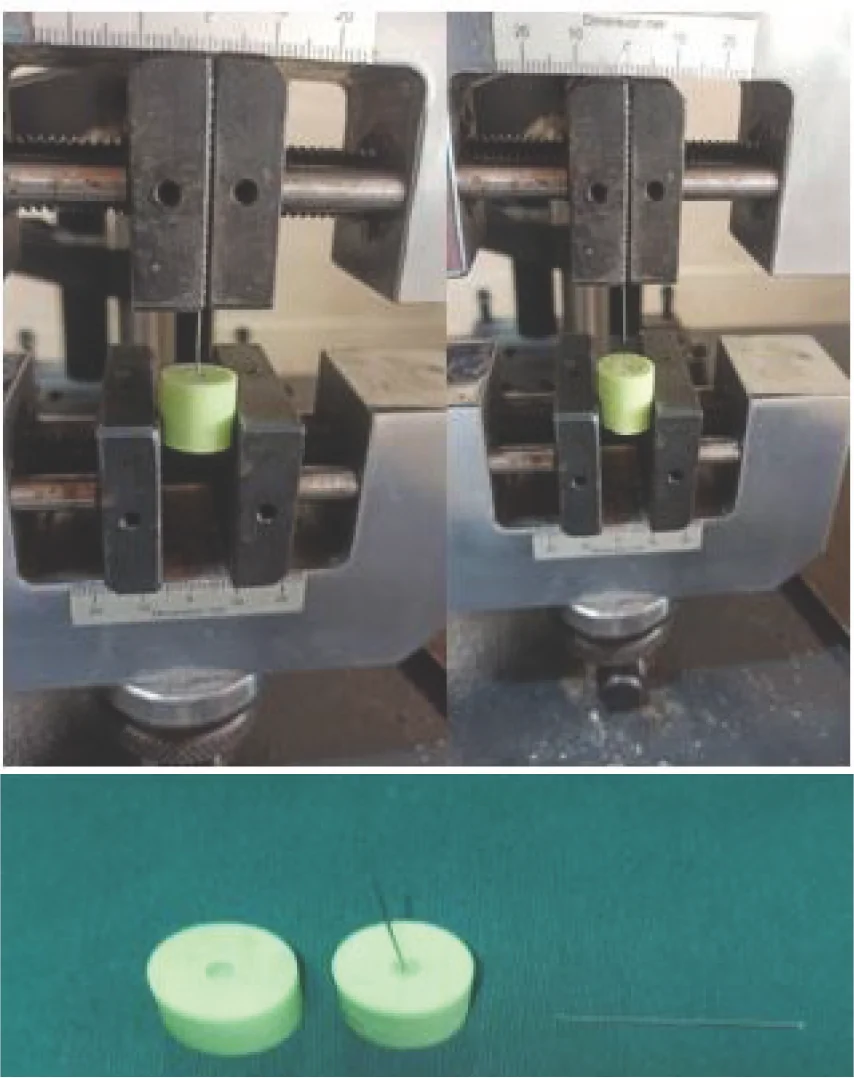
The instruction of pull-out test

The methodology utilized for assessing the pull-out force was based on the approach described by Bearn *et al*. [ 15 ]. We constructed cylindrical acrylic blocks measuring 25mm in diameter and 10mm in height. A central hole measuring 3×2mm was drilled into each block, reflecting the dimensions of the composite material typically employed in clinical applications for lingual retainers. Each wire group was paired with 20 blocks without teeth. The drilled holes were subsequently filled with composite material, into which a segment of test wire was embedded. The placement block was equipped with a stainless steel alignment jig featuring a 1mm central hole. Prior to the testing procedure, the prepared test blocks were stored in distilled water at room temperature for duration of one day. A testing machine (Instron Corp., Norwood, MA, USA) was configured in tensile mode, with the crosshead speed adjusted to 10mm/min. The test blocks were then positioned within the machine, and force was applied along the long-axis of the wires. The force necessary to separate the wires from the composite was meticulously recorded (Figure 3). 

All statistical analyses were conducted using SPSS software (version 23.0; IBM Corp., Armonk, NY, USA). The normality of data distribution was assessed using the Kolmogorov-Smirnov test (*p*> 0.05 for all variables), and homogeneity of variances was confirmed using Levene’s test. Accordingly, one-way analysis of variance (ANOVA) was used to compare pull-out force, detachment force, and deformation across the three wire groups. Post-hoc pairwise comparisons were performed using the Least Significant Difference (LSD) test. Fracture mode, as a categorical variable, was analyzed using Fisher’s Exact Test. The significance level was set at α= 0.05 for all analyses.

To assess inter- and intra-observer reliability, 10 randomly selected specimens were retested by the same investigator (intra-observer) and a second investigator (inter-observer) one week apart. Measurements of detachment force, deformation, and fracture mode were compared.

## Results

A total of 120 specimens were tested: 60 dental double blocks (for detachment force, deformation, and fracture mode) and 60 acrylic blocks without teeth (for pull-out force), each subdivided into three groups (n= 20) based on wire type-Coaxial, Dead Soft, and Multi-Strand Retainer.

The normality of the data was assessed using the Kolmogorov-Smirnov test. This test indicated that the data followed a normal distribution (*p*> 0.05). Therefore, ANOVA was used for data analysis.

Table 1 shows the mean and standard deviation of the pull-out force in the examined groups. The present study found that the highest pull-out force, with an average of 92.92 N, was associated with the Dead Soft group, while the lowest pull-out force, with an average of 38.58 N, was related to the Multi-Strand Retainer group. The ANOVA test revealed a significant difference in pull-out force among the groups (*p*= 0.001). The post-hoc LSD test showed that this difference was significant between the Coaxial and Dead Soft groups (*p*= 0.001) as well as between the Multi-Strand Retainer and Dead Soft groups (*p*= 0.001) (Table 1).

**Table 1:** Determination and comparison of mean pull out force in extracted teeth according to wire type

Wire Types	Mean	Standard Deviation	Minimum	Maximum	*p* Value
Coaxial	65.74	10.58	46.01	92.58	0.001*
Dead soft	92.92	21.34	25.97	129.61	
Multi-strand- retainer	58.38	10.76	25.97	129.61	

* A significant difference

**Figure 4:**
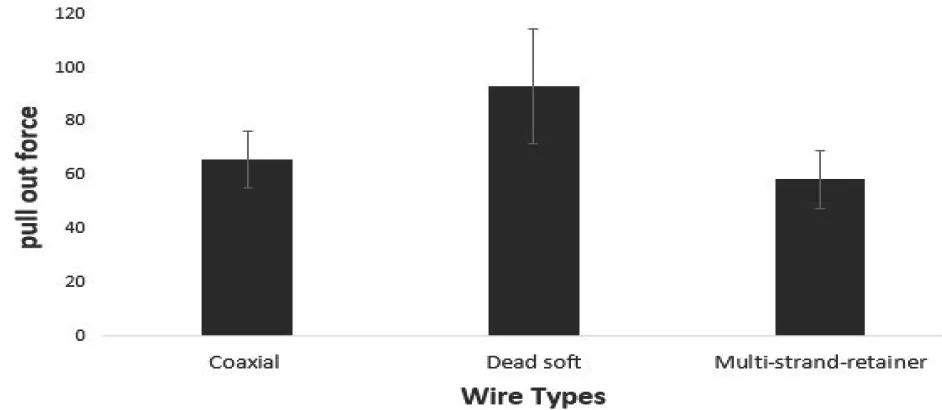
Distribution of average pull out force in extracted teeth based on wire type. Error bars represent standard deviation

Figure 4 visually represents the distribution of the mean pull-out force across the examined groups. According to this figure, the highest pull-out force is associated with the Dead Soft group, while the lowest pull-out force is related to the Multi-Strand Retainer group. 

The present study found that the highest detachment force, with an average of 51.68 N, was associated with the Dead Soft group, while the lowest detachment force, with an average of 42.75 N, was related to the Coaxial group. The analysis of variance test indicated that there was no significant difference in detachment force among the groups (*p*= 0.056) (Table 2).

**Table 2:** Determination and comparison of mean detachment force in extracted teeth according to wire type

Wire Types	Mean	Standard Deviation	Minimum	Maximum	*p* Value
Coaxial	42.75	14.56	23.10	73.82	0.056
Dead soft	51.68	11.27	36.27	83.01	
Multi-strand-retainer	49.86	10.01	32.08	70.98	

**Figure 5:**
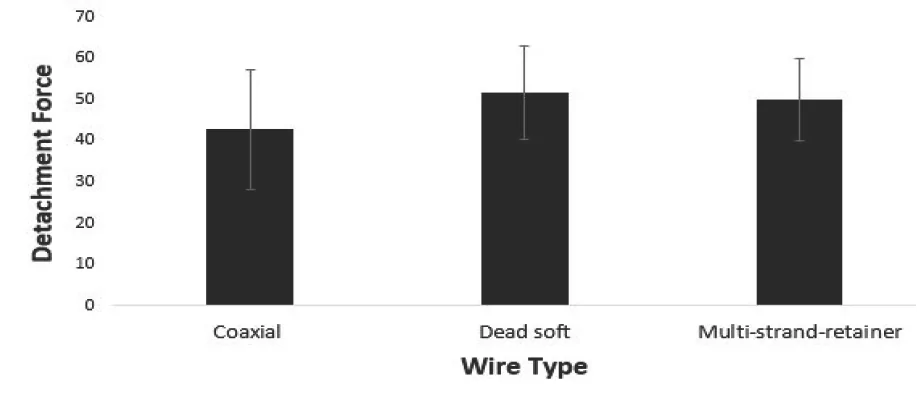
Distribution of average detachment force in extracted teeth based on wire type. Error bars represent standard deviation

Figure 5 represents the distribution of the mean detachment force across the examined groups. According to this figure, the highest detachment force is associated with the Dead Soft group, while the lowest detachment force is related to the Coaxial group. 

Table 3 shows the mean and standard deviation of deformation in the examined groups. The present study found that the highest deformation, with an average of 1.34mm, was associated with the Coaxial group, while the lowest deformation, with an average of 0.91mm, was related to the Dead Soft group. The ANOVA test indicated a significant difference in deformation among the groups (*p*= 0.001). The post-hoc LSD test revealed that this difference was significant between the Coaxial and Dead Soft groups (*p*= 0.001) as well as between the Multi-Strand Retainer and Dead Soft groups (*p*= 0.001).

**Table 3:** Determination and comparison of mean deformation in extracted teeth according to wire type

Wire Types	Mean	Standard Deviation	Minimum	Maximum	*p* Value
Coaxial	1.34	0.41	0.85	2.18	0.001*
Dead soft	0.91	0.29	0.23	1.52	
Multi-strand-retainer	1.33	0.36	0.74	2.12	

* A significant difference

**Figure 6:**
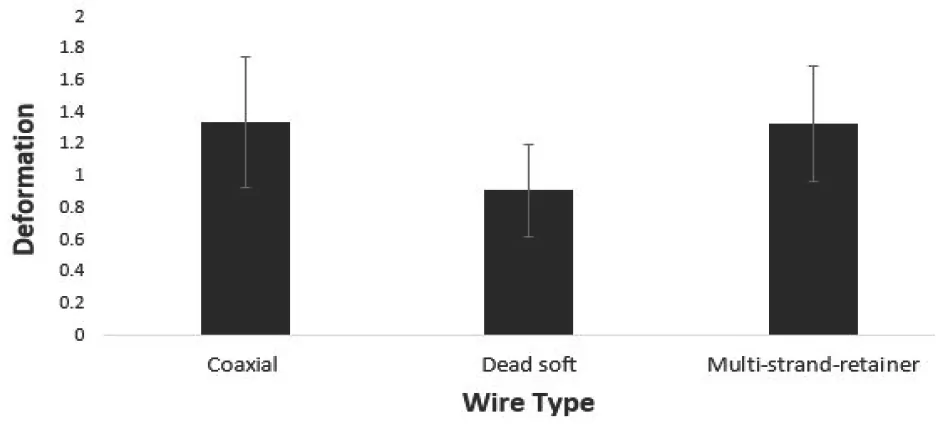
Distribution of average deformation in extracted teeth based on wire type. Error bars represent standard deviation

According to figure 6, the highest deformation is associated with the Coaxial group, while the lowest deformation is related to the Dead Soft group. 

Fracture modes were assessed by a single blinded investigator. To evaluate measurement reliability, 10 randomly selected specimens were re-evaluated one week later by the same investigator (intra-observer) and a second investigator (inter-observer). Intra-class correlation coefficients (ICC) were calculated, showing excellent agreement for both intra-observer (ICC= 0.94) and inter-observer (ICC = 0.91) reliability.

Table 4 shows the frequency distribution of fracture types in the examined groups. In this study, fracture types were categorized as follows: 0 (no composite remaining on the enamel), 1 (less than 50% of composite remaining on the enamel), 2 (more than 50% of composite remaining on the enamel), and 3 (all composite remaining on the enamel surface). The present study found that the highest incidence of type three fractures was associated with the Coaxial wire, occurring in 90% of cases, while no type three fractures were reported for the Dead Soft wire. The exact chi-square test indicated that the distribution of fracture types varied according to the type of wire (*p*= 0.001). 

**Table 4:** Frequency distribution of fracture types in extracted teeth according to wire type

Wire Types	1: Number (Frequency)	2: Number (Frequency)	3: Number (Frequency)	Total
Coaxial	0 (0)	2 (10)	18 (90)	20(100)
Dead soft	2 (10)	18 (90)	0 (0)	20(100)
Multi-strand-retainer	0 (0)	16 (80)	4 (20)	20(100)
Total	2 (3.3)	36 (60)	22 (36.7)	60(100)

## Discussion

The current investigation sought to evaluate the mechanical properties of three distinct types of wires utilized in the fabrication of BLRs, with a particular emphasis on detachment force, deformation, fracture mode, and pull-out force. The findings offer significant insights into the efficacy of these wires, which play a crucial role in orthodontic retention. 

The pull-out force represents a vital mechanical characteristic in orthodontics, especially concerning BLRs. It quantifies the strength necessary to detach the wire from the composite material. This metric is fundamental for assessing the longevity and dependability of retainers, which are intended to preserve tooth alignment over prolonged durations [ 16 - 17 ]. An increased pull-out force signifies a more robust bond between the wire and the composite, thereby diminishing the likelihood of retainer failure and minimizing the necessity for frequent repairs or replacements. It is imperative to comprehend the elements that affect pull-out force, including wire type, surface treatment, and bonding techniques, to enhance the efficacy of BLRs and ensure sustained stability in orthodontic treatment results [ 16 - 17 ].

In our study, the pull-out force, which quantifies the strength necessary to remove the wire from the composite material, was found to be greatest in the Dead Soft group (92.92 N) and least in the Multi-Strand Retainer group (58.38 N). The Coaxial wire demonstrated a moderate pull-out force of 65.74 N. These findings are in agreement with earlier studies that have assessed the mechanical attributes of various wire types. Annousaki *et al*. [ 1 ] compared fiber-reinforced composite (FRC) wires with stainless steel multistranded wires, revealing that FRC wires exhibited superior pull-out forces due to enhanced bonding capabilities with composite materials. Likewise, Alhakim *et al*. [ 5 ] indicated that Dead Soft wires, owing to their flexibility and superior adaptation to the tooth surface, generally display higher bond strengths compared to stiffer wires such as multistranded retainers. The elevated pull-out force recorded in the Dead Soft group in our study can be attributed to its capacity to closely conform to the tooth surface, thereby increasing the contact area for bonding. Conversely, the Multi-Strand Retainer group exhibited the lowest pull-out force, which is consistent with observations made by Bearn *et al*. [ 15 ], who noted that while multistranded wires are flexible, they often possess lower bond strengths due to their intricate surface geometry, which can impede optimal adhesion. The Coaxial wire, characterized by its intermediate pull-out force, exemplifies a compromise between flexibility and rigidity. Sifakakis *et al*. [ 13 ] emphasized that Coaxial wires offer moderate bond strength while retaining adequate flexibility for clinical applications.

The detachment force is a key mechanical parameter in orthodontics, as it measures the force required to separate the wire from the tooth surface. Detachment force reflects the resistance of the bonded assembly to separation at the enamel–composite–wire interface, providing insight into the clinical durability of the retainer under functional stresses [ 18 ]. In our study, the detachment force was recorded as highest in the Dead Soft group (51.68 N) and lowest in the Coaxial group (42.75 N). The Multi-Strand Retainer group exhibited a detachment force of 49.86 N. These findings are consistent with earlier research that has investigated the bond strength of various wire types. Kavousinejad *et al*. [ 4 ] analyzed the shear bond strength of ribbon and twisted wire retainers, concluding that wires with superior surface adaptation, such as Dead Soft wires, demonstrated enhanced bond strengths. This observation aligns with our results, where the Dead Soft group displayed the highest detachment force, likely attributable to its capacity to closely conform to the tooth surface, thereby improving the bond. The Coaxial group, which recorded the lowest detachment force, may illustrate the difficulties encountered when bonding rigid wires to the tooth surface. This assertion is supported by Gökçe *et al*. [ 8 ], who noted that while rigid wires provide stability, they frequently exhibit lower bond strengths due to their limited ability to adapt to the tooth surface compared to more flexible wires. The intermediate detachment force observed in the Multi-Strand Retainer group corresponds with the findings of Meade *et al*. [ 10 ], who indicated that multistranded wires, despite their flexibility, can display variable bond strengths influenced by surface treatment and bonding protocols.

The analysis of deformation indicated that the Coaxial group experienced the greatest deformation at 1.34mm, whereas the Dead Soft group recorded the least deformation at 0.91mm. The Multi-Strand Retainer group demonstrated a deformation of 1.33mm. These findings align with earlier research investigating the mechanical properties of orthodontic wires. For example, Vaida *et al*. [ 11 ] assessed the deformation characteristics of various wire types and concluded that Dead Soft wires, owing to their enhanced flexibility, typically exhibit reduced deformation under stress compared to their more rigid counterparts. This observation is consistent with our results, where the Dead Soft group displayed minimal deformation, highlighting its capacity to endure stress without considerable bending. The Coaxial group, which showed the highest level of deformation, underscores the inherent rigidity associated with this type of wire, rendering it more susceptible to bending when subjected to stress. This observation is corroborated by Sifakakis *et al*. [ 13 ], who remarked that while rigid wires offer stability, they are also more vulnerable to deformation under mechanical forces. The deformation observed in the Multi-Strand Retainer group was comparable to that of the Coaxial group, likely due to the intricate geometry of multistranded wires, as discussed by Bearn *et al*. [ 15 ] the authors indicated that the presence of multiple strands in these wires can result in uneven stress distribution, contributing to increased deformation.

The analysis of fracture modes demonstrated notable variations among the different wire types. The Coaxial group displayed the highest occurrence of type 3 fractures (90%), where all composite material remained adhered to the enamel surface, signifying a robust bond between the wire and the composite. Conversely, the Dead Soft group recorded no type 3 fractures, with the majority classified as type 2 (90%), indicating that more than 50% of the composite remained on the enamel. The Multi-Strand Retainer group exhibited a combination of type 2 (80%) and type 3 (20%) fractures. The predominance of ARI score 3 in the Coaxial group suggests that bond failure occurred primarily at the wire–composite interface, whereas the absence of score 3 in the Dead Soft group indicates that debonding involved the enamel–composite interface or cohesive failure within the composite. These results align with prior research investigating the fracture modes associated with various wire types. For instance, Sowmya *et al*. [ 14 ] assessed the ARI of different lingual retainers and discovered that rigid wires, such as coaxial wires, typically demonstrate elevated ARI scores, which suggest stronger adhesion to the composite material. This observation is corroborated by our findings, where the Coaxial group exhibited a high frequency of type 3 fractures, indicative of a strong bond with the composite. The absence of type 3 fractures in the Dead Soft group implies a weaker bond between the wire and the composite, which corresponds with its increased pull-out and detachment forces. This phenomenon may be attributed to the wire's flexibility, allowing it to deform under stress, resulting in bond failure at the wire-composite interface rather than at the enamel-composite interface. This assertion is supported by Kartal *et al*. [ 3 ], who observed that while flexible wires offer improved adaptation to the tooth surface, they may also demonstrate reduced bond strengths due to their propensity to deform under stress.

The fracture mode observed in the Multi-Strand Retainer group, characterized by a combination of type 2 and type 3 fractures, reflects the intricate geometry of these wires, which can result in varying bond strengths. This observation is consistent with the findings of Quinzi *et al*. [ 7 ], who noted that multistranded wires can display diverse fracture modes contingent upon the surface treatment and bonding protocols employed.

The evaluation of fracture modes using the ARI provides valuable insights into the nature of bond failure, which has direct consequences for both orthodontic practice and patient care. By knowing the bond strength to wire and enamel and composite cohesive strength in the tests, we can make better and more accurate decisions about composites.

This study has several limitations. Although 120 extracted teeth were used, the sample size per group (n= 20) may limit the generalizability of the findings to a broader population. Moreover, the *in vitro* design cannot fully replicate the dynamic oral environment- including saliva, thermal cycling, occlusal forces, and pH variations- nor does it assess long-term performance under cyclic loading or material degradation. The mechanical tests were conducted under controlled laboratory conditions, which do not simulate the complex biomechanical and biological challenges of the clinical setting. Consequently, the durability and failure modes of these retainers over extended periods remain to be evaluated *in vivo*.

Future research should prioritize clinical trials to evaluate the long-term performance of Coaxial, Dead Soft, and Multi-Strand Retainer wires in actual patients, with monitoring of debonding rates, wire breakage, and periodontal health over time. Additionally, surface characteristics of these wires- such as roughness and topography- should be analyzed using techniques like scanning electron microscopy (SEM) or profilometry to better understand their influence on bonding strength. Studies employing larger and more diverse tooth samples, accounting for variations in enamel quality, patient age, and storage conditions, would enhance external validity. Finally, to isolate the effect of material composition from geometric differences, future *in vitro* investigations should compare wires matched for cross-sectional area or diameter.

## Conclusion

In conclusion, the findings of this study hold significant clinical relevance for the selection of wires used in BLRs. The Dead Soft wire, characterized by its substantial pull-out and detachment forces alongside minimal deformation, is deemed an appropriate option for scenarios necessitating both flexibility and robust bonding. Nonetheless, its propensity for type 2 fractures indicates the necessity for meticulous attention to bonding conditions to reduce the likelihood of bond failure. Conversely, the Coaxial wire, which demonstrates considerable deformation and a strong bond- evidenced by a high occurrence of type 3 fractures- may be better suited for situations where rigidity and stability are essential. However, its vulnerability to deformation under stress raises concerns regarding its suitability for patients experiencing high occlusal forces or those susceptible to bruxism. The Multi-Strand Retainer wire presents an intermediate performance profile concerning pull-out force, detachment force, and deformation, thereby providing a compromise between flexibility and rigidity. Nevertheless, its inconsistent fracture modes necessitate careful evaluation of the bonding protocol to ensure reliable performance.
